# Association Between the Oral Health Status and Sociodemographic Factors Among 5–15-Year-Old Schoolchildren from Mallorca, Spain—A Cross-Sectional Study

**DOI:** 10.3390/children12040527

**Published:** 2025-04-20

**Authors:** Daniela Vallejos, Irene Coll, Nora López-Safont

**Affiliations:** 1 Faculty of Dentistry, University ADEMA University School, C. Passamaners 11, 07009 Palma, Spain; d.vallejos@eua.edu.es (D.V.); i.coll@eua.edu.es (I.C.); 2 Health Group, University Institute for Research in Health Sciences (IUNICS), Ctra. Valldemossa Km 7.5, 07122 Palma, Spain; 3 Biology Department, University of Balearics Islands, Ctra. Valldemossa Km 7.5, 07122 Palma, Spain

**Keywords:** caries, schoolchildren, sociodemographic, periodontal disease

## Abstract

*Background:* Oral health is a key indicator of general health, well-being, and quality of life. Sociodemographic factors can affect children’s oral health status. The aim of this study was to analyze the sociodemographic factors that influence the oral health of schoolchildren in Mallorca. *Materials and methods:* We conducted a cross-sectional observational epidemiological study in Mallorca, analyzing different indicators of oral health, such as the DMFT/dmft index and the Community Periodontal Index (CPI), and sociodemographic variables among 718 schoolchildren aged 5–6, 12 and 15 years. *Results:* The DMFT (Decayed, Missing, and Filled Permanent Teeth) caries index was higher in public (Pub) schools than in private/charter (P/C) schools for children in the sixth grade of elementary school (Pub, 0.6918 ± 1.272; P/C, 0.323 ± 0.824; *p* < 0.05) and in the fourth year of secondary school (Pub, 1.178 ± 1.724; P/C, 0.627 ± 1.195; *p* < 0.05), as determined using a *t*-test. First-grade elementary students with more highly educated mothers/guardians had a lower rate of DMFT (Decayed, Missing, and Filled Primary Teeth) caries than those whose mothers obtained only elementary-level education (higher, 0.800 ± 1.616; elementary, 3.333 ± 3.393; *p* < 0.05). Regarding periodontal health, we observed that sixth-grade elementary schoolchildren with more highly educated mothers/guardians had more healthy sextants (higher, 3.987 ± 1.977; elementary, 1.333 ± 2.461; *p* < 0.001). *Conclusions:* The sociodemographic and parental factors analyzed, such as the type of school and parents’ education levels, significantly affected the oral health of the schoolchildren in this study.

## 1. Introduction

Oral health is a key indicator of general health, well-being, and quality of life. The World Health Organization (WHO) defines oral health as “the absence of chronic orofacial pain, mouth or throat cancer, oral infections and ulcers, periodontal disease, caries, tooth loss, and other diseases or disorders that limit the affected person’s ability to bite, chew, smile and speak, while impacting their psychosocial well-being” [1]. It has been estimated that oral diseases affect at least 3.5 billion people worldwide, with dental caries and periodontal disease being the most prevalent of all the diseases assessed [2].

Nationally, the Oral Health Survey 2020 conducted in Spain indicates that the percentage of 5–6-year-old children in elementary school with a history of caries affecting the primary dentition is 35.5%; at 12 and 15 years of age, the prevalence in permanent dentition is 28.6% and 35.5%, respectively [3]. The prevalence in the adult cohort ranges from 93.8% to almost 100% [3]. In the Balearic Islands, an oral health survey involving schoolchildren carried out in 2005 estimated that the percentage of individuals with a history of caries in the primary dentition at 7 years of age was 45%; at 12 and 14 years of age, the prevalence in permanent dentition was 35% and 60.2%, respectively [4]. Given that nearly two decades have passed since the last data were made available, updating this information is essential to accurately assess oral health trends and identify the most pressing need among the child population.

Dental caries is a complex disease expressed as a series of factors interacting simultaneously. Its variables are of different orders, ranging from biological processes to complex historical–cultural structures and social relations, socioeconomic level, and education level, among others, making this issue even more complex [5].

According to the WHO, there is a strong and consistent association between socioeconomic status (income–occupation–education level of caregivers) and the prevalence and severity of illness in schoolchildren. This association exists throughout life, from early childhood to older age, in populations in high-, middle-, and low-income countries [6]. Socioeconomic status constitutes a critical factor affecting children’s oral health, partly serving as an indicator of a family’s ability to respond to dental problems and influencing caregivers’ perceptions when assessing their children’s oral health needs [7].

The factors associated with social context have been explained within the framework of the social determinants of health, defined as “the circumstances in which people are born, grow up, work, live and grow old, including the broader set of forces and systems that influence the conditions of everyday life”. These will condition a series of variables, including health-related practices and access to health care [8,9,10].

Socioeconomic circumstances can influence children’s habits through their caregivers’ knowledge and behaviors. The oral-health-related behaviors studied in this regard include hygiene habits, nutritional habits, and regular dental check-ups. The key elements that impact children’s oral health behavior and status are parental oral-health-related attitudes, general knowledge, and health status [11,12,13]. Specifically, these differences are reflected in a higher prevalence and risk of dental caries and periodontal disease in groups of children of low socioeconomic status, with a strong effect on oral health during childhood and later in life [14,15].

The appearance of caries lesions and other oral diseases is not the only domain in which inequalities emerge. The use of health centers and attendance at regular preventive dental check-ups are also affected by social variables. Adults who do not receive dental treatment are typically from lower socioeconomic backgrounds [16].

Geographical location is also a determining factor because it conditions access to health and education systems, public transportation, and stores. In the case of rural areas, this is manifested in unique economic, social, and structural characteristics that precipitate social gaps and increase social inequalities in health [17]. For example, adults in rural areas visit the dentist less often, have cleanings less often, and undergo more extractions of permanent teeth than adults in urban areas [18].

Another factor to consider is a child’s country of origin. A study conducted in Sweden showed that this factor is decisive in caries development; 50% of immigrant children had caries, while this figure was 20% for children born in Sweden [19]. Several studies reaffirm the influence of the differential factor between the immigrant and native populations [11,20].

This study aimed to analyze the sociodemographic factors that influence the oral health of schoolchildren in Mallorca. Based on previous literature and population health data, we tested the null hypothesis that sociodemographic variables are not significantly associated with oral health outcomes in this population.

## 2. Materials and Methods

### 2.1. Study Design

Based on a cross-sectional observational epidemiological study conducted on the school-attending population of Mallorca between October 2018 and December 2019, the association between oral health indicators, brushing frequency, urgency of intervention, perception of oral health, and sociodemographic variables (sex, place of birth, type of school, geographic location and caregivers’ education level) was analyzed. The methodology of this study was designed in accordance with the WHO recommendations for conducting oral health surveys in the manual Oral Health Surveys: Basic Methods [21].

The sample size was determined as follows. Based on a population of 12,000 children and a caries prevalence of 0.35, as reported by the National Institute of Statistics, a minimum of approximately 340 children was required to achieve a 95% confidence level with a 5% margin of error. The design, protocol, and methodology of the cross-sectional study are described in more detail elsewhere [22].

The information on the schools was extracted from the Directorate General of Planning, Organization, and Centers of the Autonomous Community of the Balearic Islands (CAIB) and the National Statistics Institute (INE).

Systematized and stratified random sampling was used. The sample was grouped into 3 strata according to the characteristics of the population of Mallorca (Table 1).

The final sample was composed of 718 students grouped into the 3 reference age cohorts (index ages) recommended by the WHO, i.e., 5–6 years (n = 255), 12 years (n = 230), and 15 years (n = 233); these students were recruited from the 28 schools selected using the stratified cluster sampling technique.

### 2.2. Data Collection

Fieldwork was conducted between November 2018 and December 2019.

The seven examining dentists performed the inspections under standardized conditions, using a headlamp (Petzl, Crolles, France), an intraoral flat mirror #5 (Schmidt, Móstoles, Spain), and a WHO periodontal probe (Derby, Lucca, Italy), according to the WHO recommendations for oral health surveys [21].

The WHO recommendations were followed for the calibration and training of the examining dentists. The training, which included theoretical sessions and practical exercises on caries detection and periodontal assessment, was followed by a four-day monitoring conference, as detailed in Coll et al. [22], in accordance with the WHO methodology [21]. To ensure standardization and reproducibility across examiners, interexaminer agreement was assessed using Cohen’s Kappa coefficient. The percentage of simple agreement was 98.7%, and the Kappa index determined using the Landis and Koch [23] scale was 0.757, which signifies a high level of agreement (greater than 95% and greater than 0.61 for Kappa) and is considered adequate for beginning a study.

The data analyzed were collected using the form defined by the WHO for oral health surveys (Annex 2 of Oral Health Surveys: Basic Methods) and the oral health questionnaire for children (Annex 8 of Oral Health Surveys: Basic Methods) [21].

### 2.3. Ethical Data Handling and Statistical Analysis

Before it was conducted, this study was approved by the ethics committee of the Balearic Islands (CEI: IB3737/18) as per current legislation and conducted in accordance with the principles outlined in the Declaration of Helsinki and the standards of good clinical practice. Before data were collected, an information sheet and an informed consent form were given to the caregivers; only children who submitted a consent form signed and dated by their parents or guardians participated in this study.

All information collected was entered into an automated file with restricted access, and personal data were encrypted to ensure confidentiality.

The data were analyzed with the SPSS 27.0.1.0^®^ (IBM Corp., Armonk, NY, USA) statistics application depending on the variables to be studied and diagnostic criteria. To compare means, a Student’s *t*-test or a one-way analysis of variance (ANOVA) was used in conjunction with a Bonferroni post hoc analysis. We compared percentages using the chi-square test via the cross-table procedure. Pearson’s bivariate correlation analysis was employed to analyze correlations in parametric data.

In each case, to make an accurate measurement (of the random error present in the data), the 95% confidence interval estimate was used (*p* < 0.05).

### 2.4. Variables to Be Studied and Diagnostic Criteria

The sociodemographic variables were as follows:

- Age;
- Sex;
- Type of school—public or private/charter;
- Geographical location—urban or rural;
- Education level of parents/guardians—elementary, secondary, or higher education.

The oral health variables were as follows:

- State of the dentition. A lesion presenting as an unmistakable cavity on the tooth surface was considered caries, as per the WHO 5th edition criteria [21]. Decayed (D), missing (M), and filled (F) teeth were recorded to calculate the prevalence of caries;
- DMFT index for primary dentition—the average of the sum of teeth with caries and filled teeth of all the schoolchildren examined (measured at 5–6 years old);
- DMFT index for permanent teeth—the mean of the sum of the numbers of teeth with caries, teeth absent due to caries, and filled teeth of all the schoolchildren examined (measured at 5–6, 12 and 15 years old);
- Prevalence of caries—the percentage of individuals with treated and active caries lesions (dmft/DMFT > 0), and the percentage of schoolchildren with active caries lesions (c/C > 0);
- Restorative index—the ratio of the total number of filled teeth to the total index under study (DMFT for permanent teeth or dmft for primary teeth), multiplied by 100. RI = [FT/(DMFT or dmft)] × 100;
- Bratthal’s SiC Index (Significant Caries Index). This is defined as the mean DMFT obtained from the third of the sample distribution with the highest caries scores. This was used as a complement to the DMFT;
- Sealed teeth. Sealants are considered a preventive intervention;
- Periodontal status was measured with the community periodontal index (CPI) and the number of healthy sextants in the 12- and 15-year-old cohorts. Six sites from each of the index teeth (16, 11, 26, 31, 36, and 46) were explored with the WHO periodontal probe and assessed as healthy (0), bleeding (1), or presenting dental calculus (2), recording only the highest value for each tooth;
- Urgency of intervention. This is determined according to the presence of caries, periodontal disease, or any other type of complication derived from them (0 = no treatment, 1 = preventive treatment, 2 = early treatment, and 3 = treatment for infection or pain);
- Frequency of brushing—determined via responses to the survey question (Never (0), <than once a day (1), once a day (2), 2 or more times per day (3), or NR (No Response)/DK (Don’t Know)) (4);
- Perception of health status. This was derived from answers to the question how would you describe the health of your teeth? 1 = excellent; 2 = very good; 3 = good; 4 = fair; 5 = poor; 6 = very poor; 9 = I do not know.

## 3. Results

### 3.1. Description of the Study Sample

During the period in which the study was conducted, 718 students were examined. These were 5–6-year-olds from the first year of elementary school (mean age: 6.08 ± 0.45 years), 12-year-olds from the sixth year of elementary school (mean age: 11.20 ± 0.56 years), and 15-year-olds from the fourth year of compulsory secondary school (mean age: 15.32 ± 0.72 years). Table 2 shows the distribution of the schoolchildren according to age, sex, and type of school.

### 3.2. Variables Related to Caries Disease

Caries disease was analyzed by assessing the influences of different sociodemographic variables on caries indicators, and the findings of this analysis are detailed below.

The results according to sex, country of birth, and type of school are given in Table 3, and have been differentiated for primary and permanent dentition.

- Primary dentition

In the 5–6 years age group, girls were observed to have worse oral health than boys, with a higher prevalence of caries (46.8% vs. 34.7%, *p* = 0.044) and active caries (38.7% vs. 25%, *p* = 0.019) and a higher caries index in dmft primary teeth (the mean of the sum of primary teeth with caries and filled) (1.819 ± 2.580 vs. 1.1250 ± 2.301; *p* = 0.024).

A comparison of the mean number of sealed primary teeth revealed that the schoolchildren in this cohort who attended public schools had fewer sealed teeth than those who attended private/charter schools (Pub: 0.006 ± 0.08; P/C: 0.07 ± 0.31, *p* = 0.014).

- Permanent dentition

The 5–6-year-old cohort exhibited a higher prevalence of caries in girls, 11.7%, than in boys, 4.9% (*p* = 0.044). At 5–6 years of age, the eruption of the permanent teeth begins, establishing mixed dentition; therefore, at this age both dentitions are valued. In the 12-year-old group, children from public schools had worse oral health statuses than those from private/charter schools, reflected in the greater prevalence of active caries (15.1% vs. 4.2%, *p* = 0.018) and the higher DMFT index (0.6918 ± 1.272 vs. 0.323 ± 0.824; *p* = 0.026).

A similar result was observed in the 15-year-old group, wherein the type of school (public or private/charter) also affected oral health. The children in public school exhibited a higher prevalence of caries (48.9% vs. 30.2%, *p* = 0.026) and a higher DMFT index (Pub, 1.178 ± 1.724; P/C, 0.627 ± 1.195, *p* = 0.048).

Furthermore, there was an association between school location and oral health status in this group, with schoolchildren attending urban schools having better oral health than those from rural areas (prevalence of caries, 32.7 vs. 55.3%; *p* < 0.001; DMFT index—R, 1.386 ± 1.860 and U, 0.673 ± 1.225; *p* < 0.001). This outcome may be related to the fact that in this group, the proportion of parents/guardians who attained higher education was higher among the individuals from urban areas compared to that for children from rural areas (urban, 80.4%; rural, 55.6%), and the same was true for mothers/guardians (urban, 79.5%; rural, 61%).

The SiC index (Significant Caries Index), corresponding to the DMFT index in the third of the sample with the highest caries index in their permanent dentition, was influenced by school location (urban/rural). In the 5–6-year-old cohort, the index was higher in urban areas than in rural areas (urban, 0.695 ± 0.973; rural, 0.193 ± 0.673, *p* = 0.009), contrasting with what was observed for the 15-year-old cohort, for which this index was higher in rural areas than in urban areas (rural, 3.156 ± 1.880; urban, 2.293 ± 1.353, *p* = 0.039). This index was also influenced by the type of school (public or private/charter)—in the 12-year-old cohort, it was higher for individuals in public school than for those in private/charter school (Pub, 1.963 ± 1.465; P/C, 1.045 ± 1.214; *p* = 0.011).

Regarding the restorative index, which provides information on access to dental care, we noted that in the 12-year-old group, this index was lower for public school students compared to that for private/charter school students (Pub, 56.802 ± 6.824; P/C, 87.500 ± 7.752, *p* = 0.026).

The influence of the guardian’s education level on caries and restorative indicators can be seen in Table 4, which has been differentiated for primary and permanent dentition.

- Primary dentition

**Figure 1:**
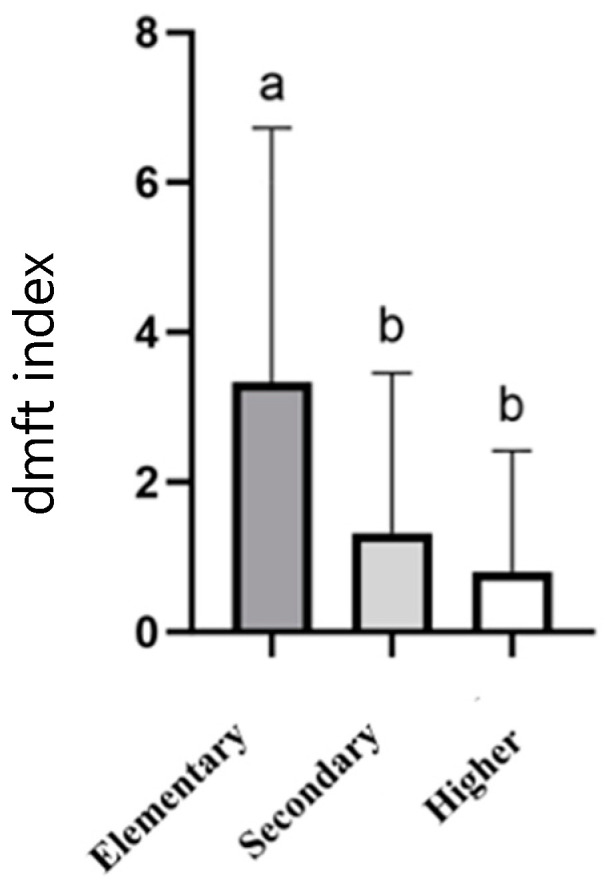
dmft index for 5–6-year-old schoolchildren according to their guardians’ education levels. The results are presented as mean ± SD. Columns that do not share the same letter (a,b) indicate a significant difference (ANOVA, *p* < 0.05, and Bonferroni post hoc analysis).

For the 6-year-old group, an association was observed between the dmft index and the mother/guardian’s education level, with the index increasing as the mother/guardian’s education level decreases (elementary, 3.333 ± 3.393; secondary, 1.322 ± 2.135; and higher, 0.800 ± 1.616; <0.001), as shown in Figure 1. An analysis of the father’s education level did not show a significant influence (see Supplementary Table S1).

- Permanent dentition

**Figure 2:**
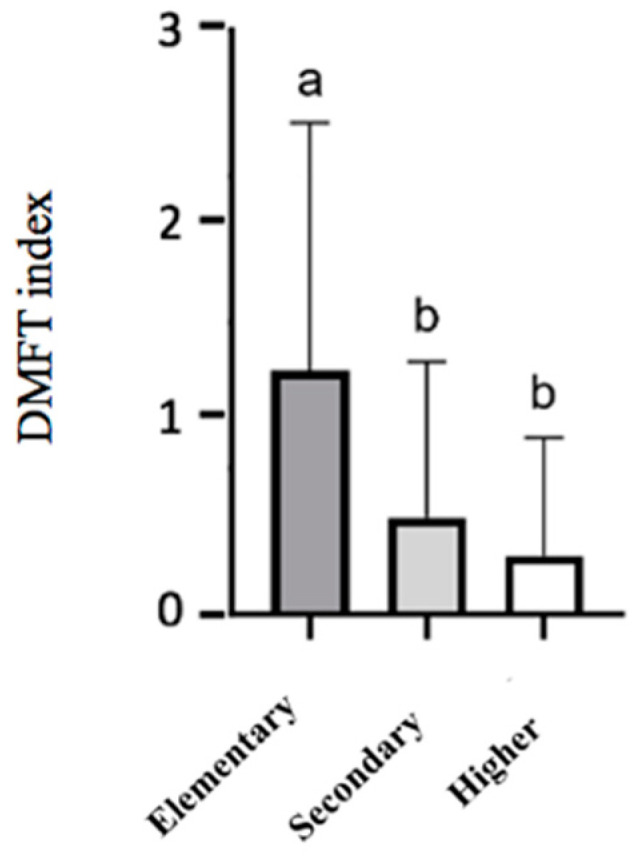
DMFT index for 12-year-old schoolchildren according to their guardians’ education levels. The results are presented as mean ± SD. Columns that do not share the same letter (a,b) indicate a significant difference (ANOVA, *p* < 0.05, and Bonferroni post hoc analysis).

In the 12-year-old cohort, an effect of the mother/guardian’s education level on some indicators was observed. The data analysis revealed a higher prevalence of caries in schoolchildren whose mothers have lower education levels (elementary, 50%; secondary, 32.5%; higher, 18.8%; *p* = 0.033), a greater frequency of active caries (elementary, 41.7%; secondary, 7.5%; higher, 5.9%; *p* < 0.001), and a greater DMFT index (elementary, 1.333 ± 1.669; secondary, 0.650 ± 1.166; higher, 0.364 ± 0.884, *p* = 0.010), as shown in Figure 2. These differences were not observed when analyzing the influence of the father/guardian’s education level (see Supplementary Table S1).

The data on the parents/guardians’ education levels for the students in their fourth year of secondary school show that active caries were more prevalent among students with less educated parents (elementary, 28.6%; secondary, 16.4%; higher, 6.52%, *p* = 0.011) (see Supplementary Table S1).

### 3.3. Variables Related to Periodontal Disease

Periodontal status was only assessed in the cohorts of sixth-grade elementary school and fourth-year secondary school students, according to the guidelines provided by the WHO.

Upon analyzing the mean number of periodontally healthy sextants in the 12-year-olds, we did not observe significant differences between the groups according to sex, type of school, and geographic location, unlike what was observed in the 15-year-olds, for whom we noted better periodontal health among students from rural schools than urban schools if we considered the mean number of healthy sextants (urban, 2.584 ± 2.196; rural, 3.282 ± 2.228, *p* = 0.020) and periodontally affected sextants (urban, 3.366 ± 2.230; rural, 2.515 ± 2.236, *p* = 0.004) (see Supplementary Table S2).

At 12 and 15 years of age, there is a trend towards a higher mean number of healthy sextants in girls, although this is not statistically significant.

**Figure 3:**
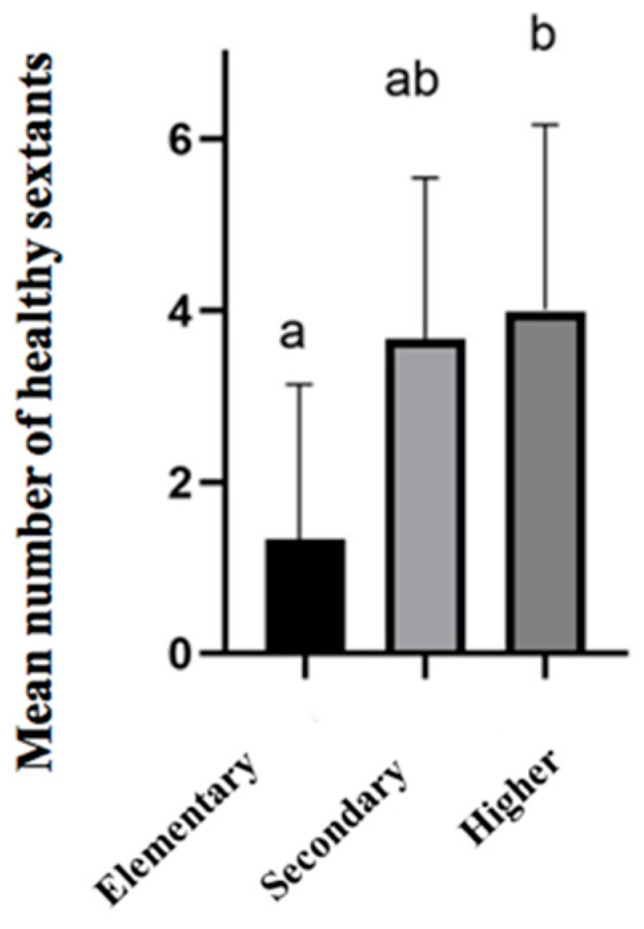
Mean number of healthy sextants in 12-year-old schoolchildren according to their guardians’ education levels. The results are presented as the mean ± SD. Columns that do not share the same letter (a,b) indicate a significant difference (ANOVA, *p* < 0.05, and Bonferroni post hoc analysis).

On the other hand, the results suggest that a lower education level of the mother/guardian is associated with a worse periodontal status among children (see Supplementary Table S3). When we evaluated the mean number of healthy sextants, this association was found to be significantly lower for children whose mothers have only completed elementary school (1.333 ± 2.461) compared to those whose mothers have completed secondary school (3.675 ± 2.055, *p* = 0.002) or underwent higher education (3.987 ± 1.977, *p* < 0.001) (Figure 3). In terms of parents/guardians, we found no association between these variables (see Supplementary Table S4).

### 3.4. Analysis of the Urgency of Intervention

This indicator provides information on how urgently children require dental treatment (see Supplementary Table S5), indicating that girls in the 5–6-year age group have a greater need for early treatment than boys (27.9% vs. 13.9%; *p* = 0.016).

In the 12-year-old age group, public school students required early treatment more urgently than private/charter school students (15.7% vs. 5.6%; *p* < 0.001).

In the 12-year-old cohort, the mother/guardian’s education level is an important factor that affects a child’s need for immediate treatment (see Supplementary Table S6), i.e., their oral health status). The need for early treatment decreases as the mother’s education level increases (elementary, 33.3%; secondary, 17.5%; higher, 5.9%; *p* = 0.024). The father’s education level does not seem to influence this variable (see Supplementary Table S7).

### 3.5. Analysis of Brushing Frequency

When analyzing the results obtained from the question regarding the frequency of brushing (see Supplementary Table S7), it was observed that the type of school affected the results for the 5–6-year-old cohort, with a higher percentage of private/charter school students brushing two or more times a day compared to that for public school students (44.4% vs. 33.3%; *p* = 0.023).

In the 12-year-old group, the guardian’s education level was a determining factor when assessing hygiene habits (see Supplementary Tables S8 and S9). The proportion of children who brush two or more times a day was higher among children whose mothers/guardians have a higher level of education (elementary, 41.7%; secondary, 57.5%; higher, 67.9%; *p* = 0.025). The same association was observed when associating this variable with the education level of the fathers/guardians (elementary, 40.9%; secondary, 64.7%, higher, 68%; *p* = 0.003).

### 3.6. Analysis of Oral Health Perceptions

In the analysis of the schoolchildren’s perception of dental health, the influence of the mother/guardian’s education level (see Supplementary Table S11) was found to be significant, showing that more children aged 5–6 years whose mothers/guardians have higher education levels consider themselves to have excellent dental health compared to children whose mothers have lower education levels (higher, 42.2% vs. secondary, 16.7%; *p* = 0.028). A similar situation was observed for 15-year-old schoolchildren, for whom a greater proportion with good oral health have mothers/guardians with higher education levels (higher, 21.9%; secondary, 9.3%; *p* = 0.034). The results are not significant when associating this variable with the fathers’ education levels (see Supplementary Table S10).

## 4. Discussion

Analyses of the data obtained in this study indicated that sociodemographic factors influenced the oral health indicators of the schoolchildren who participated in this project.

The findings of this study concerning caries disease indicate that in the case of the 5–6-year-old cohort, the prevalence of caries was higher in girls than in boys for both primary and permanent dentition, and girls also required more urgent dental treatment. This difference has already been described in a previous survey conducted in the Balearic Islands [4], but in the 14-year-old cohort, girls had a higher prevalence of caries. The influence of sex was also observed regarding periodontal status. At 12 years of age, girls showed a higher prevalence of gingival bleeding compared to boys. This suggests that factors not analyzed in this study, such as nutritional or hormonal influences, may be playing a role, especially since no significant difference was found in brushing frequency between the two groups.

When we analyzed the oral health indicators by school type, differences between students attending public and private/charter schools were observed. These differences were reflected in various aspects such as caries prevalences, DMFT index, access to dental care, and periodontal health. One factor that could partly explain these disparities is the frequency of brushing, which we observed was higher among children from private/charter schools in comparison to those from public schools.

An association between oral health and type of school has been observed in another study [24], and it has been reported that children from public schools had significantly higher values for indicators such as the mean number of decayed teeth and gingival bleeding [24].

Although we do not have all the data required to directly determine the socioeconomic levels of the schoolchildren who participated in this study, if we analyze the data provided by the National Institute of Statistics [25], they indicate that private or charter schools are always concentrated in high-income areas, in contrast to public schools, which are mainly located in areas with fewer resources, with a predominance of low- and lower-middle-class students [26]. This gives us an idea of the socioeconomic profiles of the children attending each type of school.

Another determining factor is geographic location; for the 15-year-old age group, residing in urban areas was associated with a lower prevalence of caries and a lower DMFT index than in rural areas. This may be related to the fact that in this group, the proportion of caregivers with higher levels of education is greater in urban areas than in rural areas. These results are consistent with those from previous studies conducted in other countries such as Croatia [27], Chile [28], and Russia [29], where worse oral health was also observed in rural areas, which could be explained by factors such as limited access to oral health care, low levels of education, and less healthy lifestyles. However, the periodontal health of 15-year-old students was better in rural areas than in urban areas, so other factors associated with their diets or nutritional statuses may be involved in this disease.

The SiC is an indicator that reflects the oral situation of the population most affected by caries; with this in mind, in our study, the highest values were concentrated in rural areas among students 15 years of age, in public schools among students 12 years of age, and among foreign-born schoolchildren 5–6 years of age. This difference has also been observed in studies conducted in Costa Rica [30] and Saudi Arabia [31], where the SiC for public schools was significantly higher than that for private schools. It is important to include this index in epidemiological studies because it focuses on the part of the population most affected by dental caries, and, in practice, it helps one select the most vulnerable groups on which education and health care programs should be focused.

Our analysis suggests that the education level of the mother/guardian has a strong influence on oral health indicators compared to that of the father/guardian. Specifically, a lower level of maternal education is linked to poorer oral health outcomes in children, including a higher rate of caries and reduced periodontal health. On the other hand, a better perception of the dental health of schoolchildren aged 5–6 and 12 years is associated with a higher educational level of the mother/guardian. These findings are consistent with those from previous studies, such as Spain’s last oral health survey in 2020 [3], which also observed better oral health among children whose caregivers had a higher social level, and described the effect of social status on the need for treatment.

Additionally, oral hygiene habits, particularly brushing frequency, appear to be influenced by the caregiver’s education, with higher education levels associated with more frequent brushing among children.

Some limitations of this study should be considered. The cross-sectional design limits the ability to establish causal relationships, as there was no follow-up. While various sociodemographic factors were analyzed, there may be other variables not considered that could influence oral health outcomes. Furthermore, self-reported data on oral hygiene habits could introduce biases, as responses may not always reflect actual behaviors. Despite these limitations, the study provides valuable insights into the factors influencing oral health in schoolchildren.

To complement these results, we consider it necessary to evaluate other aspects that impact oral health and that may also be conditioned by sociodemographic variables, such as habits related to a child’s diet and nutritional status.

## 5. Conclusions

The results indicate that social environment variables, especially the education level of the mother, significantly influence the prevalence of caries and periodontal disease, as well as the oral hygiene habits of the schoolchildren evaluated. The type of school attended also plays an important role, with students in public showing worse oral health outcomes compared to those in private/charter schools. Geographic location also influences oral health indicators; adolescents in urban areas have fewer caries than those in rural areas.

Although oral health indicators have improved in recent years, caries and periodontal disease are still considered high-prevalence diseases and continue to affect individuals with a low socioeconomic status more frequently, as shown in the results of this study. These findings underscore the complex interactions of various factors in the development of oral diseases, and highlight the need for targeted promotion and prevention strategies aimed at vulnerable groups.

## Figures and Tables

**Table 1:** Description of the different strata that comprise the sample.

First Stratum	Population Centers	Urban Rural
Second stratum	School types	Public Charter/Private
Third stratum	Age groups	5–6 years old (1st-grade elementary) 12 years old (6th-grade elementary) 15 years old (4th-year secondary)

**Table 2:** Distribution of the sample by grade, sex, and type of school.

		1st Grade Elementary (5–6 Years Old) n = 255		6th Grade Elementary (12 Years Old) n = 230		4th Year Secondary (15 Years) n = 233		Total	
		n	%	n	%	n	%	n	%
Sex	Male	144	56.47	125	54.35	112	48.06	381	53.06
	Female	111	43.53	105	46.65	121	51.93	337	46.94
Type of school	Public	177	69.4	159	69.1	190	81.5	526	73.3
	Private/Charter	78	30.6	71	30.9	43	18.5	192	26.7
Geographic location	Urban	163	63.9	140	60.9	101	43.3	404	56.3
	Rural	92	36.1	90	39.1	132	56.7	314	43.7

(n, sample size).

**Table 3:** Caries prevalence, restorative index, and SiC index according to sex, country of birth, and type of school.

	n	Prevalence of Caries (%)		Prevalence of Active Caries Lesions (%)		Caries Index (Dmft/DMFT ± SD)		Restorative Index (RI ± SE)		Significant Caries Index (SiC ± SD)	
5–6 years old, primary	255		*p*-Value (Chi-square)		*p*-Value (Chi-square)		*p*-Value (Student’s *t*)		*p*-Value (Student’s *t*)		*p*-Value (Student’s *t*)
Boys	144	50 (34.7%)	0.044 *	36 (25%)	0.019 *	1.1250 ± 2.301	0.024 *	36.748 ± 6.355	0.563	-	-
Girls	111	52 (46.8%)		43 (38.7%)		1.819 ± 2.580		31.833 ± 5.620		-	-
Public school	177	77 (43.5%)	0.085	61 (34.5%)	0.070	1.598 ± 2.536	0.092	33.292 ± 4.796	0.694	-	-
Private school/charter	78	25 (32.1%)		18 (23.1%)		1.038 ± 2.194		37.171 ± 8.985		-	-
Urban school	163	61 (37.4%)	0.264	46 (28.2%)	0.205	1.380 ± 2.477	0.130	35.267 ± 5.457	0.769	-	-
Rural school	92	41 (44.6%)		33 (35.9%)		1.510 ± 2.401		32.718 ± 6.723		-	-
5–6 years old, permanent	255										
Boys	144	7 (4.9%)	0.044 *	6 (4.2%)	0.068	0.0833 ± 0.4345	0.086	21.4286 ± 14.869	0.848	0.212 ± 0.689	0.132
Girls	111	13 (11.7%)		11 (9.9%)		0.198 ± 0.629		17.948 ± 10.415		0.473 ± 0.892	
Public school	177	14 (7.9%)	0.953	11 (6.2%)	0.663	0.152 ± 0.597	0.385	23.809 ± 11.284	0.408	0.338 ± 0.871	0.851
Private school/charter	78	6 (7.7%)		6 (7.7%)		0.087 ± 0.329		8.333 ± 8.333		0.300 ± 0.470	
Urban school	163	16 (9.8%)	0.119	14 (8.6%)	0.101	0.171 ± 0.614	0.124	17.708 ± 8.801	0.736	0.695 ± 0.973	0.009 *
Rural school	92	4 (4.3%)		3 (3.3%)		0.065 ± 0.324		25.000 ± 25.000		0.193 ± 0.673	
12 years old	230										
Boys	125	31 (24.8%)	0.336	13 (10.4%)	0.491	0.552 ± 1.167	0.710	65.053 ± 8.165	0.810	1.815 ± 1.486	0.498
Girls	105	32 (30.5%)		14 (13.3%)		0.609 ± 1.1643		62.239 ± 8.316		1.589 ± 1.427	
Public school	159	49 (30.8%)	0.081	24 (15.1%)	0.018 *	0.6918 ± 1.27	0.026 *	56.802 ± 6.824	0.026 *	1.963 ± 1.465	0.011 *
Private school/charter	71	14 (19.7%)		3 (4.2%)		0.323 ± 0.824		87.500 ± 7.752		1.045 ± 1.214	
Urban school	140	36 (25.7%)	0.477	14 (10%)	0.307	0.4857 ± 1.042	0.133	65.740 ± 7.656	0.676	1.900 ± 1.295	0.341
Rural school	90	27 (30%)		13 (14.4%)		0.722 ± 1.324		60.802 ± 8.971		1.574 ± 1.542	
15 years old	233										
Boys	112	47 (42%)	0.298	14 (12.5%)	0.827	1.084 ± 1.831	0.978	72.553 ± 6.221	0.366	3.114 ± 2.111	0.249
Girls	121	59 (48.8%)		14 (11.6%)		1.074 ± 1.472		79.548 ± 4.762		2.651 ± 1.395	
Public school	190	93 (48.9%)	0.026 *	25 (13.2%)	0.260	1.178 ± 1.724	0.048 *	76.379 ± 4.109	0.963	2.871 ± 1.809	0.854
Private school/charter	43	13 (30.2%)		3 (7%)		0.627 ± 1.195		76.923 ± 10.764		2.75 ± 1.281	
Urban school	101	33 (32.7%)	<0.001 *	8 (7.9%)	0.093	0.673 ± 1.225	<0.001 *	80.303 ± 6.424	0.500	2.293 ± 1.353	0.039 *
Rural school	132	73 (55.3%)		20 (15.2%)		1.386 ± 1.860		74.703 ± 4.744		3.156 ± 1.880	

(n, sample size; SD, standard deviation; SE, standard error; *, variable with a significant effect).

**Table 4:** Prevalence of caries, caries index, and restorative index according to the guardian/mother’s education level.

	n	Prevalence of Caries (%)		Prevalence of Active Caries Lesions (%)	*p*	Caries Index Dmft/DMFT ± SD	*p*	Restorative Index RI ± SE	*p*
5–6 years old, primary	255	%	*p*-Value (Chi-square)		*p*-Value (Chi-square)		*p*-Value (ANOVA)		*p*-Value (ANOVA)
1. Elementary	12	8 (66.7%)		6 (50%)		3.333 ± 3.393		40.71 ± 14.64	
2. Secondary	31	14 (45.2%)	0.50	10 (32.3%)	0.093	1.322 ± 2.135	0.001 *	28.571 ± 12.529	0.562
3. Higher	50	15 (30%)		10 (20%)		0.800 ± 1.616		46.944 ± 12.256	
Unknown	125	-	-	-	-	-	-	-	-
5–6 years old, permanent	255								
1. Elementary	12	1 (8.3%)		1 (8.3%)		0.083 ± 0.288		0	-
2. Secondary	31	2 (6.5%)	0.793	1 (6.5%)	0.793	0.0645 ± 0.249	0.969	0	-
3. Higher	50	2 (4%)		2 (4%)		0.060 ± 0.313		0	-
Unknown	125	-	-	-	-	-	-	-	-
12 years old	230								
1. Elementary	12	6 (50%)		5 (41.7%)		1.333 ± 1.669		27.777 ± 18.087	
2. Secondary	40	13 (32.5%)	0.033 *	3 (7.5%)	<0.001 *	0.650 ± 1.166	0.010 *	76.923 ± 12.162	0.055
3. Higher	85	16 (18.8%)		5 (5.9%)		0.364 ± 0.884		75.000 ± 10.206	
Unknown	72	-	-	-	-	-	-	-	-
15 years old	233								
1. Elementary	8	3 (37.5%)		0		0.750 ± 1.164		100	
2. Secondary	55	31 (56.4%)	0.103	10 (18.2%)	0.083	1.363 ± 1.637	0.196	72.688 ± 7.378	0.438
3. Higher	143	57 (39.9%)		12 (8.4%)		0.916 ± 1.629		79.678 ± 5.028	
Unknown	21	-	-	-	-	-	-	-	-

(n, sample size; SD, standard deviation; SE, standard error; * variable with a significant effect).

## Data Availability

The datasets generated and/or analyzed during the current study are not publicly available as they are being utilized for ongoing purposes; however, they can be made available by the corresponding author upon reasonable request.

## Supplementary Materials

The following supporting information can be downloaded at https://www.mdpi.com/article/10.3390/children12040527/s1. Table S1. Prevalence of caries, caries index, and restoration according to the guardian/father’s education level. Table S2. Mean number of affected and healthy sextants according to sex, type of school, and geographic location of the schoolchildren. Table S3. Mean number of healthy and affected sextants according to the mother/guardian’s education level. Table S4. Mean number of healthy and affected sextants according to father/guardian’s education level. Table S5. Urgency of intervention according to sex, type of school, and geographic location of the schoolchildren. Table S6. Urgency of intervention according to the mother/guardian’s level of education. Table S7. Frequency of brushing according to sex, type of school, and geographic location of the schoolchildren. Table S8. Frequency of brushing according to the mother/guardian’s education level. Table S9. Frequency of brushing according to father/guardian’s education level. Table S10. Perception of oral health according to the father/guardian’s education level. Table S11. Perception of oral health according to the mother/guardian’s education level.

## Abbreviations

The following abbreviations are used in this manuscript:

WHO	World Health Organization
FAO	Food and Agriculture Organization of the United Nations
EFSA	European Food Safety Authority
DMFT	Decayed (D), Missing (M), and Filled (F) for permanent Teeth.
dmft	Decayed (D), Missing (M), and Filled (F) for primary teeth.
