# Supplementary material for: Association Between the Oral Health Status and Sociodemographic Factors Among 5–15-Year-Old Schoolchildren from Mallorca, Spain—A Cross-Sectional Study
Source: Children (Basel). 2025 Apr 20;12(4):527. doi: 10.3390/children12040527 (PMC12025421; doi:10.3390/children12040527)
Supplement: Supplementary file 1 [file children-12-00527-s001.zip › children-3521990-supplementary.pdf]

Supplementary tables

Table 1. Prevalence of caries, caries index and restoration according to the guardian/father's education level.

	n	Prevalence of Caries (%)		Prevalence of Active Caries Lesions (%)	*p*	Caries Index Dmft/DMFT ± SD	*p*	Restorative Index RI ± SE	*p*
5-6 years primary	255		***p* -Value (Chi-square)**		***p* -Value (Chi-square)**		**p -Value (ANOVA)**		***p* -Value (ANOVA)**
1. Elementary	11	4 (36.36%)		2 (18.18%)		0.909±1.44		50.0±28.867	
2. Secondary	38	12 (31.57%)	0.957	10 (26.3%)	0.589	1.02±2.00	0.763	23.809±11.305	0.231
3. Higher	46	15 (32.60%)		8 (17.39%)		0.760±1.33		54.4±12.436	
Unknown	126	-	-	-	-	-	-	-	-
5-6 years permanent									
1. Elementary	11	1 (9.09%)		1 (9.09%)		1.181±0.603		0	-
2. Secondary	38	1 (2.63%)	0.485	1 (2.63%)	0.485	0.026±0.162	0.141	0	-
3. Higher	46	1 (2.17%)		1 (2.17%)		0.021±0.147		0	-
Unknown	126	-	-	-	-	-	-	-	-
12 years	230								
1. Elementary	22	8 (36.4%)		5 (22.72%)		0.812±1.367		54.166±16.592	
2. Secondary	34	6 (17.64%)	0.254	2 (5.88%)	0.061	0.264±0.618	0.053	66.666±21.081	0.684
3. Higher	72	16 (22.22%)		5 (6.94%)		0.3611±0.774		71.875±11.151	
Unknown	81	-	-	-	-	-	-	-	-
15 years	233								
1. Elementary	14	8 (57.14%)		4 (28.6%)		1.28±1.589		56.250±17.519	
2. Secondary	55	28 (50.90%)	0.186	9 (16.4%)	**0.011***	1.018±1.394	0.728	73.333±7.615	0.155
3. Higher	138	54 (39.13%)		9 (6.5%)		0.942±1.642		82.716±4.810	
Unknown	24	-	-	-	-	-	-	-	-

**Figures in bold indicate a significant difference between variables.*

Table 2. Mean number of affected and healthy sextants according to sex, type of school, and geographic location of the schoolchildren.

12 years	n	mean±SD Affected	***p* -Value (Student's t)**	n	mean±SD Healthy	***p* -Value (Student's t)**
Boys	123	1.902±1.985	0.229	120	3.891±2.069	0.051
Girls	104	2.240±2.231		99	3.313±2.288	
Public school	156	2.109±2.180	0.584	157	3.586±2.295	0.635
Private/charter school	71	1.943±1.933		62	3.741±1.889	
Urban school	139	2.000±2.176	0.607	130	3.523±2.289	0.382
Rural school	88	2.147±1.991		89	3.786±2.025	
15 years						
Boys	112	3.125±2.313	0.119	111	2.693±2.283	0.067
Girls	121	2.661±2.211		121	3.239±2.228	
Public school	190	2.831±2.82	0.459		3.021±2.229	0.548
Private/charter school	43	3.11±2.216			2.797±2.177	
Urban school	101	3.366±2.230	**0.004***	101	2.584±2.196	**0.020***
Rural school	132	2.515±2.236		131	3.282±2.228	

**Figures in bold indicate a significant difference between variables.*

Table 3. Mean number of healthy and affected sextants according to the mother/guardian's education level					
12 years	n	mean±SD Affected	*p* -Value (ANOVA)	mean±SD Healthy	*p* -Value (ANOVA)
1. Elementary	11	3.181±2.786		1.333±2.461	
2. Secondary	33	2.181±2.007	0.262	3.675±2.055	**0.002***
3. Higher	59	1.9153±1.958		3.987±1.977	
15 years					
1. Elementary	8	2.500±2.563		3.500±2.563	
2. Secondary	54	3.222±2.279	0.446	2.581±2.298	0.399
3. Higher	137	2.868±2.232		2.298±2.240	

**Figures in bold indicate a significant difference between variables.*

Table 4. Mean number of healthy and affected sextants according to father/guardian's education level					
12 years	n	mean±SD Affected	*p* -Value (ANOVA)	mean±SD	*p* -Value (ANOVA)
1. Elementary	17	2.352±2.396		3.00±2.533	
2. Secondary	29	1.931±2.136	0.753	4.00±2.047	0.170
3. Higher education	57	2.175±1.992		3.956±1.973	
15 years					
1. Elementary	14	3.142±2.769		2.428±2.709	
2. Secondary	53	2.584±2.299	0.277	3.381±2.392	0.213
3. Higher education	132	3.075±2.177		2.832±2.161	

Table 5. Urgency of intervention according to sex, type of school, and geographic location of the schoolchildren.					
	n	No treatment required	Preventive treatment	Early treatment/ Immediate treatment	p
5-6 years	255				*p* -Value (Chi-square)
Boys	144	103 (71.5%)	21 (14.6%)	20 (13.9%)	**0.016***
Girls	111	63 (56.8%)	17 (15.3%)	31 (27.9%)	
Public school	177	113 (63.8%)	25 (14.1%)	39 (22%)	0.456
Private/charter school	78	53 (67.9%)	13 (16.7%)	12 (15.4%)	
Urban school	163	105 (64.4%)	25 (15.3%)	33 (20.2%)	0.950
Rural school	92	61 (66.3%)	13 (14.1%)	18 (19.6%)	
12 years	230				
Boys	125	57 (45.5%)	55 (44%)	13 (10.4%)	0.259
Girls	105	53 (50.5%)	36 (34.3%)	16 (15.2%)	
Public school	159	62 (39%)	72 (45.3%)	25 (15.7%)	**<0.001***
Private/charter school	71	48 (67.6%)	19 (26.8%)	4 (5.6%)	
Urban school	140	66 (47.1%)	56 (40%)	18 (12.9%)	0.966
Rural school	90	44 (48.9%)	35 (38.9%)	11 (12.2%)	
15 years	233				
Boys	112	35 (31.3%)	72 (64.3%)	5 (4.5%)	0.664
Girls	121	41(33.9%)	72 (59.5%)	8 (6.6%)	
Public school	189	66 (34.7%)	112 (59.5%)	11 (5.8%)	0.301
Private/charter school	43	10 (23.3%)	31 (72.2	2 (4.7%)	
Urban school	101	31 (30.7%)	66 (65.3	4 (4%)	0.496
Rural school	132	45 (34.1%)	78 (59.1	9 (6.8%)	

**Figures in bold indicate a significant difference between variables.*

Table 6. Urgency of intervention according to the mother/guardian's level of education

	n	No treatment required	Preventive treatment	Early treatment/ Immediate treatment	p
5-6 years	255				**p -Value (Chi-square)**
1. Elementary	12	5 (41.7%)	3 (25%)	4 (33.3%)	
2. Secondary	31	22 (71%)	3 (9.7%)	6 (19.4%)	0.166
3. Higher	50	37 (74%)	8 (16%)	5 (10%)	
Unknown	125	-	-		
12 years	230				
1. Elementary	12	4 (33.3%)	4 (33.3%)	4 (33.3%)	
2. Secondary	40	15 (37.5%)	18 (45%)	7 (17.5%)	**0.024***
3. Higher	85	47 (55.3%)	33 (38.8%)	5 (5.9%)	
Unknown	72	-	-		
15 years	233				
1. Elementary	8	3 (37.5%)	4 (50%)	1(12.5%)	
2. Secondary	55	15 (27.3%)	36 (65.5%)	4 (7.3%)	
3. Higher	143	43 (30.1%)	93 (65%)	7 (4.9%)	0.813
Unknown	21	-	-		

**Figures in bold indicate a significant difference between variables.*

Table 7. Frequency of brushing according to sex, type of school, and geographic location of the schoolchildren.

	n	0-1 time a week	2-6 times a week	Once a day	2 or more times a day	p	mean±SD
5-6 years						***p* -Value (Chi-square)**	
Boys	142	27 (19%)	7 (4.9%)	45 (31.7%)	63 (44.4%)	0.512	4.802±1.530
Girls	107	27 (25.2%)	7 (6.5%)	34 (31.8%)	39 (36.4%)		4.542±1.591
Public school	171	28 (16.4%)	11 (6.4%)	56 (32.7%)	76 (44.4%)	**0.023***	4.877±1.512
Private/charter school	78	26 (33.3%)	3 (3.8%)	23 (29.5%)	26 (33.3%)		4.311±1.718
Urban school	161	39 (24.2%)	7 (4.3%)	55 (34.2%)	60 (37.3%)	0.169	4.596±1.582
Rural school	88	15 (17%)	7 (8%)	24 (27.3%)	42 (47.7%)		4.863±1.509
12 years							
Boys	122	19 (15.6%)	4 (3.3%)	26 (21.3%)	73 (59.8%)	0.516	5.114±1.397
Girls	103	21 (20.4%)	6 (5.8%)	17 (16.5%)	59 (57.3%)		4.951±1.504
Public school	154	26 (16.9%)	7 (4.5%)	29 (18.8%)	92 (59.7%)	0.951	5.077±1.421
Private/charter school	71	14 (19.7%)	3 (4.2%)	14 (19.7%)	40 (56.3%)		4.957±1.506
Urban school	139	21 (15.1%)	4 (2.9%)	25 (18%)	89 (64%)	0.139	5.201±1.336
Rural school	86	19 (22.1%)	6 (7.0%)	18 (20.9%)	43 (50%)		4.779±1.582
15 years							
Boys	112	8 (7.1%)	4 (3.6%)	21 (18.8%)	79 (70.5%)	0.111	5.464±1.064
Girls	121	9 (7.4%)	3 (2.5%)	10 (8.3%)	99 (81.8%)		5.553±1.154
Public school	190	16 (8.4%)	7 (3.7%)	27 (14.2%)	140 (73.7%)	0.172	5.441±1.179
Private/charter school	43	1 (2.3)	0	4 (9.3%)	38 (88.4%)		5.814±0.663
Urban school	100	9 (6.8%)	1 (1%)	11 (10.9%)	80 (80.2%)	0.305	5.534±1.179
Rural school	132	9 (6.8%)	6 (4.5%)	20 (15.2%)	97 (73.5%)		5.492±1.059

**Figures in bold indicate a significant difference between variables.*

Table 8. Frequency of brushing according to the mother/guardian's education level							
		0-1 time a week	2-6 times a week	Once a day	2 or more times a day	p	mean±SD
5-6 years	n					***p* -Value (Chi-square)**	
1. Elementary	12	4 (33.3%)	0	3 (25%)	5 (41.7%)		4.636±1.747
2. Secondary	30	6 (20%)	2 (6.7%)	7 (23.3%)	15 (50%)	0.594	4.862±1.574
3. Higher	50	6 (12%)	4 (8%)	17 (34%)	23 (46%)		5.04±1.301
Unknown	122		-	-	-		-
12 years							
1. Elementary	12	5 (41.7%)	2 (16.7%)	0	5 (41.7%)	**0.025***	4.250±1.658
2. Secondary	40	6 (15%)	2 (5%)	9 (22.5%)	23 (57.5%)		5.050±1.484
3. Higher	84	11 (13.1%)	2 (2.4%)	14 (16.7%)	57 (67.9%)		5.291±1.312
Unknown	71		-	-	-		-
15 years							
1. Elementary	8	1 (12.5%)	0	0	7 (87.5%)		5.500±1.414
2. Secondary	55	2 (3.6%)	4 (7.3%)	6 (10.9%)	43 (78.2%)	0.308	5.600±0.914
3. Higher	143	10 (7%)	2 (1.4%)	16 (11.2%)	115 (80.4%)		5.566±1.110
Unknown	21	-	-	-	-		-

**Figures in bold indicate a significant difference between variables.*

Table 9. Frequency of brushing according to father/guardian's education level							
		0-1 time a week	2-6 times a week	Once a day	2 or more times a day	p	mean±SD
5-6 years	n					***p* -Value (Chi-square)**	
1. Elementary	11	3 (27.3%)	1 (9.1%)	2 (18.2%)	5 (45.5%)	0.703	4.333±1.870
2. Secondary	38	6 (15.8%)	4 (10.5%)	9 (23.7%)	19 (50%)		4.891±1.448
3. Higher	45	5 (11.1%)	2 (4.4%)	15 (33.3%)	23 (51.1%)		5.133±1.254
Unknown	122		-	-	-		-
12 years							
1. Elementary	22	8 (36.4%)	3 (13.6%)	2 (9.1%)	9 (40.9%)	**0.003***	4.363±1.619
2. Secondary	34	7 (20.6%)	1 (2.9%)	4 (11.8%)	22 (64.7%)		4.935±1.672
3. Higher	72	8 (11.1%)	0	15 (20.8%)	49 (68%)		5.366±1.244
Unknown	79		-	-	-		-
15 years							
1. Elementary	14	1 (7.1%)	1 (7.1%)	0	12 (85.7%)		5.571±1.157
2. Secondary	55	3 (5.5%)	1 (1.8%)	7 (12.7%)	44 (80%)	0.738	5.685±0.842
3. Higher	138	9 (6.5%)	3 (2.2%)	19 (13.8%)	107 (77.5%)		5.548±1.104
Unknown	24		-	-	-		-

**Figures in bold indicate a significant difference between variables.*

Table 10. Perception of oral health according to the father/guardian's education level							
	n	Excellent	Very Good	Good	Fair	Poor/very poor	p
5-6 years	255						***p* -Value (Chi-square)**
1. Elementary	11	2 (18.2%)	4 (36.4%)	3 (27.3%)	1 (9.1%)	1 (9.1%)	
2. Secondary	38	6 (15.8%)	13 (34.2%)	13 (34.2%)	6 (15.8%)	0	0.1.38
3. Higher	45	18 (40%)	8 (17.8%)	11 (24.4%)	4 (8.9%)	4 (8.9%)	
Unknown	125	-	-	-	-	-	
12 years							
1. Elementary	15	1 (6.7%)	3 (20%)	6 (40%)	5 (33.3%)	0	0.970
2. Secondary	34	3 (8.8%)	7 (20.6%)	14 (41.2%)	8 23.5%)	2 (5.9%)	
3. Higher	70	7 (10%)	17 (24.3%)	23 (32.9%)	18 (25.7%)	5 (7.1%)	
Unknown	88	-	-	-	-	-	
15 years							
1. Elementary	14	0	3 (21.4%)	4 (28.6%)	7 (50%)	0	0.189
2. Secondary	55	8 (14.5%)	10 (18.2%)	18 (32.7%)	18 (32.7%)	1 (1.8%)	
3. Higher	132	7 (5.3%)	36 (27.3%)	50 (37.9%)	34 (25.8%)	5 (3.8%)	
Unknown	28	-	-	-	-	-	

Table 11. Perception of oral health according to the mother/guardian's education level							
	n	Excellent	Very Good	Good	Fair	Poor/very poor	p
5-6 years							***p* -Value (Chi-square)**
1. Elementary	12	0	4 (33.3%)	5 (41.7%)	1 (8.3%)	2 (16.7%)	
2. Secondary	30	5 (16.7%)	10 (33.3%)	8 (26.7%)	6 (20%)	1 (3.3%)	**0.028***
3. Higher	49	21 (42.9%)	7 (14.3%)	11 (22.4%)	8 (16.3%)	2 (4.1%)	
Unknown	123	-	-	-	-	-	
12 years							
1. Elementary	8	0	1 (12.5%)	3 (37.5%)	4 (50%)	0	0.454
2. Secondary	38	5 (13.2%)	7 (18.4%)	13 (34.2%)	9 (23.7%)	4 (10.5%)	
3. Higher	80	8 (10%)	21 (26.3%)	26 (32.5%)	23 (28.7%)	2 (2.5%)	
Unknown	71	-	-	-	-	-	-
15 years							
1. Elementary	8	0	1 (12.5%)	2 (25%)	5 (62.5%)	0	
2. Secondary	54	6 (11.1%)	5 (9.3%)	23 (42.6%)	18 (33.3%)	2 (3.7%)	**0.034***
3. Higher	138	8 (5.8%)	44 (31.9%)	48 (34.8%)	33 (23.9%)	5 (3.6%)	
Unknown		-	-	-	-	-	-

**Figures in bold indicate a significant difference between variables.*
